# Variable *In Vitro* Efficacy of Delafloxacin on Multidrug-Resistant *Pseudomonas aeruginosa* and the Detection of Delafloxacin Resistance Determinants

**DOI:** 10.3390/antibiotics14060542

**Published:** 2025-05-25

**Authors:** András Kubicskó, Katalin Kamotsay, Péter Banczerowski, László Sipos, Dóra Szabó, Béla Kocsis

**Affiliations:** 1 Institute of Medical Microbiology, Semmelweis University, 1089 Budapest, Hungary; 2 Central Microbiology Laboratory, National Institute of Hematology and Infectious Disease, Central Hospital of Southern-Pest, 1097 Budapest, Hungary; 3 Department of Neurosurgery and Neurointervention, Semmelweis University, 1085 Budapest, Hungary; 4 HUN-REN-SU Human Microbiota Research Group, 1052 Budapest, Hungary

**Keywords:** delafloxacin, fluoroquinolone resistance, *Pseudomonas aeruginosa*, high-risk clone, QRDR, efflux pump

## Abstract

**Background:** In this study, molecular mechanisms contributing to delafloxacin resistance in *Pseudomonas aeruginosa* strains were investigated. Delafloxacin is a recently approved fluoroquinolone currently introduced to clinical applications. **Methods:** A total of 52 *P. aeruginosa* strains were collected from clinical isolates. Antimicrobial susceptibility testing was performed via broth microdilution, and the minimum inhibitory concentration (MIC) values for ciprofloxacin, levofloxacin, delafloxacin, ceftazidime and imipenem were determined. Five delafloxacin-resistant *P. aeruginosa* strains were selected for whole-genome sequencing (WGS). **Results:** MIC50 values were determined, and the following results were obtained: ciprofloxacin 0.25 mg/L, levofloxacin 0.25 mg/L and delafloxacin 1 mg/L. All five selected strains showed both extended-spectrum beta-lactamase and carbapenemase production. WGS analysis of these strains determined the sequence types (STs), namely, ST235 (two strains), ST316 (two strains) and ST395. Several mutations in quinolone-resistance-determining regions (QRDRs) were detected in all five delafloxacin-resistant *P. aeruginosa* strains as follows: *gyrA* Thr83Ile and *parC* Ser87Leu mutations were present in all five strains, while *parE* Thr223Ala in ST235, Glu459Val in ST316 and Val200Met in ST395 were detected. MexAB-OprM and MexCD-OprJ efflux pumps were uniformly present in all delafloxacin-resistant *P. aeruginosa* strains. All strains of ST235 and ST316 carried *bla*_NDM-1_ in combination with other beta-lactamases. In our study, the *in vitro* efficacy of delafloxacin is inferior compared to previous fluoroquinolones based on MIC50 values; however, MIC values of delafloxacin ranged between 0.125 and 128 mg/L in our *P. aeruginosa* collection, and 21 out of 52 strains showed susceptibility to delafloxacin. **Conclusions**: Multiple QRDR mutations combined with several efflux pumps confer delafloxacin resistance in *P. aeruginosa*. Among the different detected multidrug-resistant *P. aeruginosa* strains in this study, we also report on an NDM-1 producing *P. aeruginosa* ST316 in Hungary.

## 1. Introduction

*Pseudomonas aeruginosa* is a ubiquitous pathogen, causing both community and healthcare-associated infections. Multidrug-resistant (MDR) *P. aeruginosa* is a serious public health threat, and its clinical importance is recognized especially in intensive care units. *P. aeruginosa* can cause different difficult-to-treat infections, and in many cases the treatment options are limited to specific antibiotics. Furthermore, *P. aeruginosa* can persist in the hospital environment, and, therefore, it can cause numerous outbreaks. *P. aeruginosa* causes several types of infections, including nosocomial infections, e.g., ventilator-associated pneumonia, bloodstream infections, urinary tract infections, catheter-associated infections and surgical site infections. It is the leading cause of pneumonia in people with cystic fibrosis [1,2,3,4].

*P. aeruginosa* is a member of the ESKAPEE (*Enterococcus faecium*, *Staphylococcus aureus*, *Klebsiella pneumoniae*, *Acinetobacter baumannii*, *P. aeruginosa*, *Enterobacter* spp. and *Escherichia coli)* pathogens, a group of high-priority multidrug-resistant bacteria, as these pathogens cause a significant burden of complicated infections. These bacterial pathogens are capable of achieving the MDR phenotype through the acquisition of mobile genetic elements by horizontal gene transfer. Furthermore, these bacteria are able to develop resistance to virtually any group of antimicrobial agents, including beta-lactams and fluoroquinolones [5,6].

MDR *P. aeruginosa* strains are associated with certain high-risk clones, and these MDR clones are widespread worldwide. The global dissemination of these clones was reported in the past years, usually in nosocomial infections, in complicated systemic infections and in hospital outbreaks. The top 10 high-risk clones of *P. aeruginosa* comprise ST235, ST111, ST233, ST244, ST357, ST308, ST175, ST277, ST654 and ST298. Among these clones, different carbapenemases are detected frequently, namely, GES, KPC, FIM, GIM, IMP, NDM, SPM and VIM. Apart from carbapenem resistance, additionally, resistance to fluoroquinolones, aminoglycosides and polymyxins have been reported in these top 10 high-risk clones of *P. aeruginosa* [1].

*P. aeruginosa* has a vast palette of virulence factors, outer membrane proteins, alginate, biofilm formation, flagella, pilli, secretory systems, exotoxins, proteolytic, lipolytic-enzymes, pyocyanin and siderophores. These have important roles in the pathogenesis of *P. aeruginosa*, and among other features, biofilm formation improves the survival of *P. aeruginosa* on living and non-living surfaces in a hospital, enhancing its ability to cause outbreaks [1,7,8,9,10].

Beta-lactams, fluoroquinolones and aminoglycosides are used for the treatment of infections caused by *P. aeruginosa*. Several studies reported that fluoroquinolone monotherapy is associated with significantly improved survival rates compared to beta-lactam monotherapy for patients with *P. aeruginosa* bacteremia [1,11,12,13,14,15]. Several resistance mechanisms can be present in *P. aeruginosa*. The main antimicrobial resistance determinants consist of beta-lactamases (e.g., extended-spectrum beta-lactamase (ESBL), AmpC type beta-lactamase), aminoglycoside-modifying enzymes and efflux pumps. Fluoroquinolone resistance mechanisms can be separated into two main groups, namely, mutations in quinolone-resistance-determining regions (QRDRs) and plasmid-mediated quinolone resistance (PMQR) determinants. QRDRs contain specific nucleic acid sequences of gyrase (*gyrA* and *gyrB*) and topoisomerase IV (*parC* and *parE*) enzymes, and certain mutations in these genes lead to amino acid changes, which alter the affinity of fluoroquinolone binding. PMQR determinants are *qnr* genes (*qnrA*, *qnrB*, *qnrC*, *qnrD*, *qnrE* and *qnrVC*), aminoglycoside acetyltransferase [*aac(6′)-Ib-cr*] and *oqxAB* and *qepA* efflux pumps [16,17,18]. However, *qnrVC* occurs usually in *P. aeuginosa* MDR clones, as reported in some earlier studies [1]. MexAB, MexCD, MexEF and MexXY efflux pumps are also able to cause fluoroquinolone resistance in *P. aeruginosa*. Carbapenem-resistant *P. aeruginosa* strains are also on the rise, and the main mechanism of carbapenem resistance is the production of carbapenemases, namely, NDM, VIM and KPC [1,19]. Additionally, other mechanisms confer carbapenem resistance in *P. aeruginosa* due to the overexpression of the MexAB-OprM efflux pump, overproduction of AmpC beta-lactamase and inactivation of the OprD outer membrane protein [1,20].

In 2024, the World Health Organization listed carbapenem-resistant *P. aeruginosa* as a high-risk group pathogen; therefore, the development of new antibiotics is required for the treatment of infections caused by carbapenem-resistant *P. aeruginosa* [21]. During recent years, several new antibacterial agents were introduced into clinical practice to combat MDR bacterial infections [22].

Delafloxacin is a new fluoroquinolone that has been recently approved for clinical use in both per os and parenteral administrations. Delafloxacin has a unique chemical structure and a larger molecular surface, and it targets both gyrase and topoisomerase IV enzymes in Gram-negative and Gram-positive bacteria. Delafloxacin binds to target enzymes with more potency compared to earlier fluoroquinolones, which makes it a promising agent to treat different bacterial infections. Delafloxacin has been approved to treat different bacterial infections, namely, community-acquired pneumonia and acute skin and soft tissue infections. Additional potential indications for delafloxacin therapy are complicated urinary tract infections, diabetic foot infections, prosthetic joint infections and bacteremia. Side effects of delafloxacin are favorable compared to earlier fluoroquinolones [23,24]. It was previously demonstrated that delafloxacin possesses enhanced antibacterial efficacy in acidic environments; thus, it can be utilized in cystic fibrosis (CF) patients, which has been demonstrated in a CF sputum time–kill model study [25].

Some earlier studies analyzed the potential application of delafloxacin to treat infections caused by MDR *P. aeruginosa*, specifically in complicated urinary tract infections [26] and in pneumonia [27]. Synergistic combinations of delafloxacin with tobramycin and delafloxacin with ceftazidime/avibactam were successfully tested against *P. aeruginosa* [13].

The aims of this study were to analyze the antibacterial efficacy of delafloxacin on a collection of *P. aeruginosa* strains and to detect resistance mechanisms that confer delafloxacin resistance.

## 2. Results

### 2.1. Antimicrobial Susceptibility Testing

**Figure 1:**
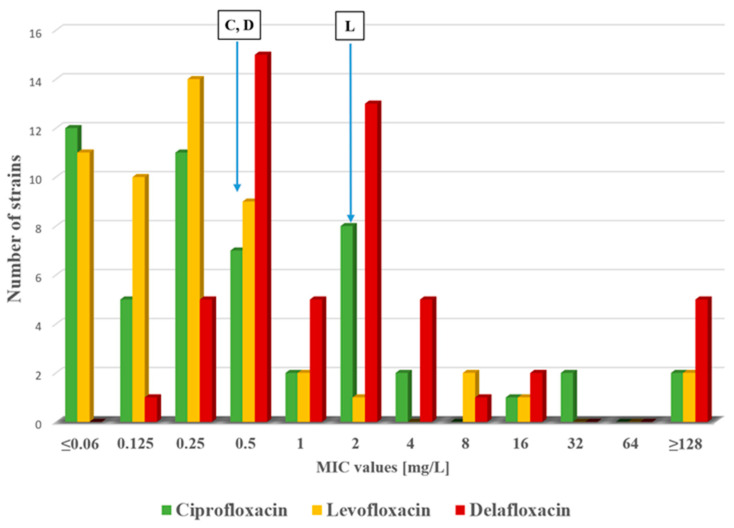
Distribution of MIC values for ciprofloxacin, levofloxacin and delafloxacin among the tested *P. aeruginosa* strains. Resistance breakpoints are shown for ciprofloxacin (C), levofloxacin (L) and delafloxacin (D). EUCAST breakpoints were used for ciprofloxacin and levofloxacin. FDA breakpoint is applied for delafloxacin.

**Figure 2:**
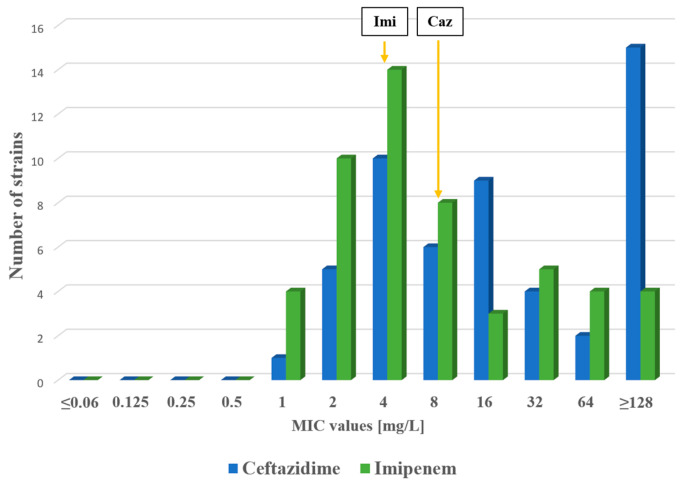
Distribution of MIC values for ceftazidime and imipenem among the tested *P. aeruginosa* strains. Resistance breakpoints are shown for ceftazidime (Caz) and imipenem (Imi). EUCAST breakpoints were used for both beta-lactams.

The minimum inhibitory concentration (MIC) values were determined for all 52 strains of *P. aeruginosa.* Susceptibility results for tested antimicrobial agents are shown in Figure 1 and Figure 2. Altogether, 35 strains were susceptible to ciprofloxacin, 47 were susceptible to levofloxacin and 21 were susceptible to delafloxacin. Thirty-one strains showed a resistant phenotype to delafloxacin. However, the distribution of delafloxacin MIC values appears to be shifted to higher values compared to other tested fluoroquinolones. We analyzed the distribution of fluoroquinolone MIC values among the 52 *P. aeruginosa* strains by a *t* test. A statistically significant difference was detected between ciprofloxacin and delafloxacin MIC values (*p* = 0.04) and between levofloxacin and delafloxacin MIC values (*p* = 0.04); however, no significant difference was found between ciprofloxacin and levofloxacin MIC values (*p* = 0.06). Simultaneous carbapenem-resistant and delafloxacin-resistant phenotypes were detected in 16 strains (30.7%). The ESBL phenotype was detected in 30 of the tested strains (57%); among these, 22 were delafloxacin resistant as well.

MIC50 values were also determined with the following results: ciprofloxacin 0.25 mg/L, levofloxacin 0.25 mg/L and delafloxacin 1 mg/L. The MIC values were interpreted based on the EUCAST recommendations for ciprofloxacin and levofloxacin. The MIC values of delafloxacin were interpreted based on the U.S. Food and Drug Administration (FDA) recommendation [23].

### 2.2. Genome Sequencing

Whole-genome sequencing (WGS) analysis was performed on five delafloxacin-resistant *P. aeruginosa* strains, and according to multi-locus sequence typing (MLST), different sequence types (STs) were detected, namely, ST235 (two strains), ST316 (two strains) and ST395 (one strain). Various types of beta-lactamase genes were detected in the analyzed *P. aeruginosa* strains: in ST235 *bla*_NDM-1_, *bla*_OXA-488_ and *bla*_PDC-35_; in ST316 *bla*_NDM-1_, *bla*_OXA-395_, *bla*_PDC-36_ and *bla*_PME-1_; and in ST395 *bla*_OXA-905_ and *bla*_PDC-8_. Additionally, other resistance determinants were also detected, and these are summarized in Table 1 and Table 2a,b.

The WGS analysis also detected relevant QRDR mutations and efflux pumps in the five tested delafloxacin-resistant strains of *P. aeruginosa*. All five of them have uniform codon changes in *gyrA* Thr83Ile and in *parC* Ser87Leu. However, in the matter of *parE*, in the ST235 strains Thr223Ala; in the ST316 strains Glu459Val and in the ST395 strain Val200Met mutations were also detected. Different efflux pump genes that have significant affinity to fluoroquinolones were found in all five *P. aeruginosa* strains. Interestingly, MexAB-OprM and MexCD-OprJ were present in all five strains; however, in ST395 and in one strain of ST235 (*P. aeruginosa* 795 strain), a MexEF-OprN was also detected. The results regarding antibiotic resistance genes are shown in Table 2a,b.

**Figure 3:**
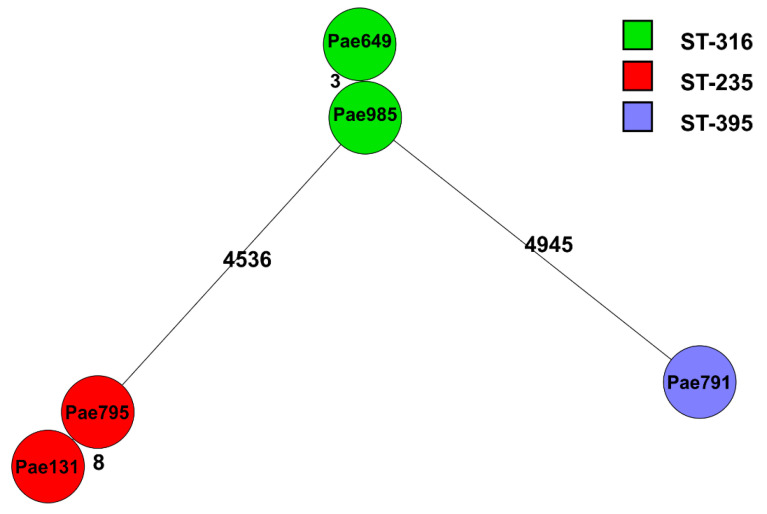
The wgMLST results of five delafloxacin-resistant *P. aeruginosa* strains. Each circle indicates a *P. aeruginosa* strain. Lines and numbers between the circles denote differences in the allelic variants between the strains, and 4536 and 4945 allelic variants indicate larger differences; however, 3 and 8 show smaller differences between the strains. Each clone is shown in a different color.

The whole-genome multi-locus sequence typing (wgMLST) results of the five delafloxacin-resistant *P. aeruginosa* strains are analyzed in Figure 3.

## 3. Discussion

*P. aeruginosa* is a prominent bacterial pathogen, a member of the ESKAPEE group that causes a high number of nosocomial infections, such as ventilator-associated pneumonia and bacteremia. Individuals with cystic fibrosis and chronic cardiorespiratory diseases are especially prone to infections caused by *P. aeruginosa.* Multidrug-resistant and carbapenem-resistant *P. aeruginosa* strains have a worldwide distribution, commonly found in clinical settings. The dissemination of resistance genes considerably narrows down the possible antimicrobial treatment options for clinicians during infections caused by MDR *P. aeruginosa* [1].

Delafloxacin is a new fluoroquinolone that was approved for multiple indications, including community-acquired pneumonia and acute skin and soft tissue infections. However, in healthcare-associated infections often caused by multidrug-resistant bacterial strains, the efficacy of this new antimicrobial agent and its impact on mortality and morbidity rates are yet to be determined.

In our study, we analyzed the distribution of MIC values in a collection of 52 *P. aeruginosa* clinical isolates. MIC50 values were 0.25 mg/L for both ciprofloxacin and levofloxacin; however, we detected 1 mg/L for delafloxacin. As shown in Figure 1, a broad distribution of delafloxacin MIC values can be seen for *P. aeruginosa* strains, ranging from 0.125 to 128 mg/L. According to the statistical analysis, a significant difference was detected between the ciprofloxacin and delafloxacin MIC value distribution as well as between levofloxacin and delafloxacin MIC values in this collection of 52 *P. aeruginosa* strains. Based on the MIC50 values, delafloxacin seems inferior to earlier fluoroquinolones; however, taking into account the delafloxacin MIC range, a certain population of *P. aeruginosa* still exhibited susceptibility to delafloxacin, making it an effective fluoroquinolone. The diversity of delafloxacin efficacy could be accounted for in the vast repertoire of antimicrobial resistance determinants (e.g., efflux pump) amongst the virulence factors, allowing the bacterium to adapt and survive in virtually any hospital setting.

The antibacterial efficacy of delafloxacin was tested on *P. aeruginosa* in an earlier study in Spain, and a 2 mg/L MIC50 value was demonstrated for delafloxacin, while MIC values of delafloxacin ranged between 0.032 and 32 mg/L in a collection of 101 MDR *P. aeruginosa* strains [28].

Five of the studied delafloxacin-resistant *P. aeruginosa* strains in this study were investigated via WGS to obtain genetical insight into the resistance mechanisms and genetic determinants as well as to analyze the clonality of the *P. aeruginosa* strains. Two of them belonged to the international high-risk clone ST235, another two were ST316 and one strain belonged to ST395.

*P. aeruginosa* ST235 is top-tier in the worldwide distributed top 10 high-risk global clones. The abundance of horizontally acquired resistance determinants, including over 60 different beta-lactamase enzymes such as ESBLs and carbapenemases, make this clone a diverse pathogen. Furthermore, infections caused by the ST235 clone are associated with higher mortality rates compared to infections of other high-risk *P. aeruginosa* clones, making this ST235 a particularly concerning pathogen [29,30,31,32,33]. Our strains of ST235 carry different antimicrobial resistance genes regarding beta-lactamases: *bla*_NDM-1_, *bla*_OXA-488_ and *bla*_PDC-35_. Among the fluoroquinolone resistance determinants, multiple QRDR mutations were detected, namely, *gyrA* Thr83Ile, *parC* Ser87Leu and *parE* Thr223Ala. In the case of efflux pumps, MexAB-OprM and MexCD-OprJ were present in both strains of ST235; however, MexEF-OprN was detected only in the *P. aeruginosa* 795 strain of ST235.

*P. aeruginosa* ST316 is a relatively uncommonly isolated clone among both hospital- and community-acquired infections, though its multidrug-resistant phenotype has already been reported [34,35]. Both strains of ST316 in our collection have multiple QRDR mutations, namely, Thr83Ile in *gyrA*, Ser87Leu in *parC* and Glu459Val in *parE*. An association between beta-lactamase production and fluoroquinolone resistance was also detected. The presence of different beta-lactamases, including *bla*_NDM-1_, *bla*_OXA-395_, *bla*_PDC-36_ and *bla*_PME-1_, verifies the observed phenotypes; essentially, 128 mg/L MIC was detected for all tested antibiotics in both strains. Among the efflux pumps, MexAB-OprM and MexCD-OprJ were present in both strains of ST316. We report this uncommon clone as a possible high-risk clone, which has all the characteristics already seen in other high-risk clones, as it possesses different beta-lactamase enzymes, aminoglycoside-modifying enzymes of [*aph(3′)-IIb, aph(3′)-Ib, aph(6)-Id, ant(4′)-IIb* and *aac(3)-II*], and fluoroquinolone resistance determinants, and all of these genes of resistance determinants are readily available for this clone; however, the actual expression rates and ratios of these are yet to be investigated and should be studied in future projects.

*P. aeruginosa* ST395 is also an international high-risk clone, reported several times in clinical isolates in Europe, mostly in France and the UK [36,37]. We detected in our ST395 strain two beta-lactamase genes, namely, *bla*_OXA-905_ and *bla*_PDC-8_, and fluoroquinolone resistance determinants, namely, QRDRs: *gyrA* Thr83Ile, *parC* Ser87Leu and *parE* Val200Met. These *gyrA* and *parC* mutations match with previously studied isolates of ST395 in Europe [38]. In our study MexAB-OprM, MexCD-OprJ, and MexEF-OprN efflux pumps were also present in ST395 clone.

In all five delafloxacin-resistant *P. aeruginosa* strains, an association between beta-lactamase production and delafloxacin resistance was detected by WGS.

Delafloxacin resistance has been analyzed recently in different bacterial pathogens. Several QRDR mutations were detected in *Helicobacter pylori* and *S. aureus* strains that exhibited resistance to delafloxacin [39,40]. In our earlier studies, we investigated resistance mechanisms in delafloxacin-resistant *E. coli* and *K. pneumoniae*. Multiple QRDR mutations in combination with PMQR determinants were detected in delafloxacin-resistant *E. coli* [41,42]. In the case of delafloxacin-resistant *K. pneumoniae* strains, the QRDR mutations were associated with OqxAB and AcrAB/TolC efflux pumps [43]. It can be summarized that high-risk clones of *E. coli* and *K. pneumoniae* were commonly detected among delafloxacin-resistant strains [41,42,43].

It has been earlier established that a diverse fitness cost is detected in different clones of MDR bacterial pathogens during achieving fluoroquinolone resistance. Among the high-risk clones of *K. pneumoniae*, *E. coli* and *S. aureus*, in the fluoroquinolone-resistant strains, a retained bacterial fitness was detected in association with double Serine mutations in QRDRs (e.g., Serine to Leucine and Serine to Isoleucine), which are considered as beneficial mutations [44]. Taking into account that *P. aeruginosa* possesses a Threonine in position 83 of Gyrase and a Serine in position 87 of Topoisomerase IV enzymes, an analogue amino acid substitution in these specific regions from a polar Threonine to an apolar Isoleucine and a polar Serine to an apolar Leucine can also correlate to retained fitness of *P. aeruginosa*. Interestingly, in our study, the *gyrA* Thr83Ile and *parC* Ser87Leu substitutions were uniformly present in all five delafloxacin-resistant strains of *P. aeruginosa* ST235, ST316 and ST395, indicating that delafloxacin resistance can commonly occur among high-risk clones of *P. aeruginosa.*

Several limitations of this study should be considered. Firstly, the sample size is only 52 *P. aeruginosa* isolates, those were acquired from one laboratory, namely South-Pest Central Hospital, National Institute of Hematology and Infectious Diseases. Second, WGS data were analyzed and interpreted, but a functional validation of efflux pump expression was not performed.

## 4. Materials and Methods

### 4.1. Strains

Our study included 52 nonrepetitive clinical isolates of *P. aeruginosa.* These were collected between September and December 2022 at South-Pest Central Hospital, National Institute of Hematology and Infectious Diseases, Budapest, Hungary. All *P. aeruginosa* strains of this study were collected from different clinical samples, such as blood culture, urine and sputum. All isolates were analyzed during routine microbiological laboratory work, and the selection of strains was conducted according to inclusion criteria. The routine identification of *P. aeruginosa* isolates was performed via matrix-assisted laser desorption ionization time-of-flight mass spectrometry (MALDI Biotyper, Bruker, Bremen, Germany). The inclusion criteria of *P. aeruginosa* strains were resistance to ciprofloxacin and/or resistance to third-generation cephalosporins or ESBL positivity by a double-disk synergy test.

### 4.2. Investigation of Minimum Inhibitory Concentration (MIC) Values

Antibiotic susceptibility testing was performed on all *P. aeruginosa* strains of this study. The broth microdilution method was applied to determine the MIC values for the following antibacterial agents: delafloxacin, ciprofloxacin, levofloxacin, ceftazidime and imipenem. We performed the microdilution method in 96-well microplates with Muller–Hinton broth. Interpretation of MIC results for ciprofloxacin, levofloxacin, ceftazidime and imipenem was performed based on the latest EUCAST protocol v15.0 (www.eucast.org (accessed on 1 January 2025)). However, the interpretation of delafloxacin MIC values was performed according to U.S. Food and Drug Administration (FDA) recommendations [23]. The control strain in this study was *P. aeruginosa* ATCC 27853 (www.atcc.org/products/27853) (accessed on 12 January 2025). The differences in the fluoroquinolone MIC values in the collection of the 52 *P. aeruginosa* strains were analyzed by the *t* test statistical program.

### 4.3. Whole-Genome Sequencing (WGS)

WGS analysis was conducted in this study on five *P. aeruginosa* strains; these were selected to detect genetic markers and resistance determinants. *P. aeruginosa* strains that exhibited delafloxacin and ciprofloxacin resistance as well as imipenem resistance and an ESBL phenotype were selected for WGS. The Illumina MiSeq platform was applied to perform WGS with a Eurofins BIOMI Kft (Gödöllő, Hungary). In all five *P. aeruginosa* strains of this study, genomic DNA was extracted by a NucleoSpin Microbial DNA Mini kit (Macherey-Nagel, Düren, Germany). The extracted DNA was measured by a qubit fluorometer and microcapillary electrophoresis (Tape Station 4150, Agilent, Waldbronn, Germany), which was applied to analyze the quality of the extracted DNA. Library preparation was performed by an Illumina DNA Prep kit (San Diego, CA, USA). Sequencing was performed on an Illumina Miseq system, and 250 bp paired-end reads were generated by a MiSeq Reagent Kit v2. Genome assembly of *P. aeruginosa* strains was carried out by the SPAdes Genome assembler algorithm v3.15.3. Bionumerics v8.1 software was applied to detect and to analyze antibiotic-resistant genes in the assembled genomes [41,42,43]. The Comprehensive Antibiotic Resistance Database (CARD 4.0.0) and Resistance Gene Identifier program (CARD RGI 6.0.3) were also applied for the analysis of resistance genes at https://card.mcmaster.ca/analyze/rgi (accessed on 12 January 2025). The WGS quality metrics are shown in Table 3.

## 5. Conclusions

The diversity in antibacterial efficacy of delafloxacin against *P. aeruginosa* in our study demonstrates this bacterium’s plasticity and variety of resistance mechanisms, and, hence, the wide spectrum of its phenotypic profiles. The efficacy of delafloxacin compared to previously used fluoroquinolones such as ciprofloxacin and levofloxacin seems inferior based on the MIC50 values in our study: ciprofloxacin 0.25 mg/L, levofloxacin 0.25 mg/L and delafloxacin 1 mg/L. This can be accounted for via the many known characteristics of this species, including the overexpression of efflux pumps. However, certain populations of *P. aeruginosa* in our study (21 isolates out of 52) showed susceptibility to delafloxacin, indicating the possible therapeutic application of delafloxacin during infections of *P. aeruginosa*. However, we suggest that delafloxacin therapy should be applied cautiously.

Among the delafloxacin-resistant *P. aeruginosa* strains, multiple QRDR mutations were detected by WGS. According to genome sequence data, the *gyrA* Thr83Ile substitution and *parC* Ser87Leu substitution are common features of delafloxacin-resistant *P. aeruginosa*. Additionally, MexAB-OprM and MexCD-OprJ efflux pumps were also uniformly present in all five delafloxacin-resistant *P. aeruginosa* strains.

*P. aeruginosa*, as a ubiquitous bacterium, is well known for its accumulation of resistance mechanisms, as it can be present in different niches, such as in humans, animals and the environment. The One Health approach suggests that strict infection control measures in hospitals and limited usage of antibiotics in non-human fields are vital to slow down the rate of widespread dissemination of MDR *P. aeruginosa* clones to make novel agents in the antibiotic pipeline able to catch up [1].

Further investigations are necessary to determine efflux pump expression rates via gene expression profiling. Additionally, *in vivo* clinical data from patients treated with delafloxacin should be assessed to confirm delafloxacin activity during infections caused by *P. aeruginosa* [45,46].

## Figures and Tables

**Table 1:** Resistance determinants of five delafloxacin-resistant *P. aeruginosa* strains. MLST: multi-locus sequence typing; beta-lactamases; antibiotic resistance genes; MIC values of ciprofloxacin (cip); levofloxacin (lev); delafloxacin (del); ceftazidime (caz) and imipenem (imi) are demonstrated. All MIC values are shown in mg/L.

	MLST	Beta-Lactamases	Other Resistance Genes	cip	lev	del	caz	imi
***P. aeruginosa*** **131**	ST235	*bla*_NDM-1_, *bla*_OXA-488_, *bla*_PDC-35_	*sul1, catB7, fosA, aph(3′)-IIb*	32	8	128	128	32
***P. aeruginosa*** **649**	ST316	*bla*_NDM-1_, *bla*_OXA-395_, *bla*_PDC-36_, *bla*_PME-1_	*sul1, catB7, fosA, aph(3′)-IIb, aph(3′)-Ib, aph(6)-Id, ant(4′)-IIb, ble, aac(3)-II*	128	128	128	128	128
***P. aeruginosa*** **791**	ST395	*bla*_OXA-905_, *bla*_PDC-8_	*catB7, fosA, aph(3′)-IIb*	32	16	128	16	8
***P. aeruginosa*** **795**	ST235	*bla*_NDM-1,_ *bla*_OXA-488_, *bla*_PDC-35_	*catB7, fosA, aph(3′)-IIb*	16	8	128	128	64
***P. aeruginosa*** **985**	ST316	*bla*_NDM-1_, *bla*_OXA-395_, *bla*_PDC-36_, *bla*_PME-1_	*sul1, catB7, fosA, aph(3′)-IIb, aph(3′)-Ib, aph(6)-Id, ant(4′)-IIb, ble, aac(3)-II*	128	128	128	128	128

**Table 2:** (**a**) Five delafloxacin-resistant *P. aeruginosa* strains with fluoroquinolone resistance determinants. QRDR: quinolone-resistance-determining region, ST: sequence type. (**b**) Five delafloxacin-resistant *P. aeruginosa* strains with fluoroquinolone resistance determinants. ST: sequence type. Bold face indicates efflux pumps with major role in fluoroquinolone resistance.

**(a)**					
**ST**	ST235 *P. aeruginosa* 131	ST316 *P. aeruginosa* 649	ST395 *P. aeruginosa* 791	ST235 *P. aeruginosa* 795	ST316 *P. aeruginosa* 985
**QRDR**	*gyrA:* Thr83Ile *parC:* Ser87Leu *parE:* Thr223Ala	*gyrA:* Thr83Ile *parC:* Ser87Leu *parE:* Glu459Val	*gyrA:* Thr83Ile *parC:* Ser87Leu *parE:* Val200Met	*gyrA:* Thr83Ile *parC:* Ser87Leu *parE:* Thr223Ala	*gyrA:* Thr83Ile *parC:* Ser87Leu *parE:* Glu459Val
**(b)**					
**ST**	ST235 *P. aeruginosa* 131	ST316 *P. aeruginosa* 649	ST395 *P. aeruginosa* 791	ST235 *P. aeruginosa* 795	ST316 *P. aeruginosa* 985
**Fluoroquinolone antibiotic efflux**	***MexA, MexB, MexC, MexD*,** *MexF, MexG, MexH, MexI, MexR, MexS, MexT MexV, MexW, MexY, MexZ, **OprJ, OprM,** OprN, rsmA, soxR, CpxR, YajC, PmpM, OpmD, adeF, ParS, ParR, Typa A NfxB, nalC, nalD*	***MexA, MexB, MexC, MexD***, *MexE, MexG, MexI, MexR, MexS, MexT, MexY, MexV, MexW, **OprJ, OprM,** OprN CpxR, rsmA, PmpM, adeF, OpmD, YajC, ParS, ParR, soxR, Type A NfxB, nalC, nalD*	***MexA, MexB, MexC, MexD MexE, MexF*** *, MexG, MexH, MexI, MexR, MexS, MexT, MexV, MexW, MexY, **OprJ, OprN, OprM**, OpmD, PmpM, rsmA, soxR, ParS, YajC, ParR, Type A NfxB, nalC, nalD*	***MexA, MexB, MexC, MexD, MexE, MexF*,** *MexG, MexH, MexI, MexS, MexT, MexV, MexW, **OprJ, OprN, OprM**, OpmD, YajC, ParS, ParR, Type A NfxB, rsmA, adeF, PmpM, soxR, CpxR, nalC, nalD, MexR*	***MexA, MexB, MexC, MexD*,** *MexE, MexG, MexH, MexI, MexR, MexS, MexT, MexV, MexW, MexY, **OprJ, OprM** OprN, YajC, ParS, PmpM, adeF, ParR, CpxR, OpmD, rsmA, soxR, Type A, NfxB, nalC, nalD*

**Table 3:** Quality metrics of sequencing.

Sample Name	Average Denovo Coverage	Number of Contigs	N50 Value
***P. aeruginosa*** **131**	98	77	251 514
***P. aeruginosa*** **649**	97	92	260 809
***P. aeruginosa*** **791**	96	61	447 543
***P. aeruginosa*** **795**	96	84	226 439
***P. aeruginosa*** **985**	95	89	217 956

## Data Availability

Genomic data of all five delafloxacin-resistant *P. aeruginosa* strains were submitted to the NCBI Genbank at the following accession numbers: Bioproject, PRJNA1254648; Biosample, SAMN48116894 (*P. aeruginosa* 131 strain), SAMN48116895 (*P. aeruginosa* 649 strain), SAMN48116896 (*P. aeruginosa* 791 strain), SAMN48116897 (*P. aeruginosa* 795 strain) and SAMN48116898 (*P. aeruginosa* 985 strain). Sequence read archive accession: *P. aeruginosa* 131 strain, SRX28529958; *P. aeruginosa* 649 strain, SRX28529959; *P. aeruginosa* 791 strain, SRX28529960; *P. aeruginosa* 795 strain, SRX28529961 and *P. aeruginosa* 985 strain, SRX28529957.
